# The Relationship Between Extroversion and the Tendency to Anthropomorphize Robots: A Bayesian Analysis

**DOI:** 10.3389/frobt.2018.00135

**Published:** 2019-01-09

**Authors:** Alexandra D. Kaplan, Tracy Sanders, Peter A. Hancock

**Affiliations:** ^1^Minds in Technology/Machines in Thought Lab, Department of Psychology, University of Central Florida, Orlando, FL, United States; ^2^MITRE, Washington, DC, United States

**Keywords:** extroversion, anthropomorphism, anthropomorphization, human-robot interaction, individual differences

## Abstract

Personality variables play an important role in how individuals relate to the world around them, including how they view their peers. Such peers now include machine entities, such as robots. This study examined the relationship between the personality trait of extroversion and the tendency to view a robot as anthropomorphic in an experimental setting. To evaluate this relationship, 486 participants were required to complete measures of the big five personality traits (The Mini IPIP) and Negative Attitudes to Robots Scale (NARS). Participants then viewed videos and images of robots performing common jobs (i.e., warehouse technician, IED detection), and then rated these robots via an assessment instrument scaling anthropomorphism. A significant positive relationship between extroversion and the tendency toward anthropomorphization of the robots was found. A Bayesian regression analysis was performed, which indicated the strength of extroversion as a predictor of the tendency to anthropomorphize. We conclude that personality dimensions influence how an individual views the robot that they interact with. These findings are important, as the relationship between personality and the tendency to anthropomorphize robots is likely to influence the acceptance and use of robots.

## Introduction

In the futuristic television cartoon, The Jetsons, their home assistance robot “Rosie,” was portrayed as an accepted part of the family. In current times, disembodied voices, such as Alexa and Siri are similarly tagged with human names and attributes. In contrast, the Roomba robot vacuum cleaner does not obtain any human appellation. Why are some technologies humanized while others remain little more than inanimate tools? The answer may lie not just in the differences between the technologies, but also in the differences between their users.

As robots increasingly occupy roles previously filled by humans, there is a need to understand those factors that influence acceptance of, and confidence in them. While robot characteristics are themselves an important and well-studied factor in understanding this, characteristics of the human user are also critical in human-robot interactions (HRI), and an area that requires more study. The range of reactions to the same robot by different users, suggests that a large part of a person's perception of a robot can be explained by human individual differences rather than the characteristics of the robot *per se*. For instance, an individual's familiarity with technology is a strong predictor of their positive reactions to robots (Parasuraman and Riley, 1997; Hancock et al., 2011). However, there are also many other individual difference factors that influence reactions to robots, such as personality variables. The present study focuses particularly on the trait of introversion-extroversion, since this is a well-validated and important dimension of personality that has a strong relationship to how individuals perceive and relate to the world around them (Eysenck, 1950; Donnellan et al., 2006) including how they view machine entities, such as robots. Previous studies have demonstrated strong evidence of an association between characteristics of extroversion, such as low communication apprehension, and positive responses to automated technologies; conversely, negative attitudes toward robots were found to be associated with high levels of “communication apprehension” (Nomura et al., 2008). These findings may be explained by the tendency of extroverted individuals to demonstrate strong preferences for communication, and lower levels of communication apprehension in a number of settings. Furthermore, since extroversion is associated with anthropomorphic tendencies (Letheren et al., 2016), we anticipate that extroversion will also be strongly related to robot anthropomorphization.

An important characteristic of Human-Robot Interaction (HRI) that may be influenced by personality is the tendency to anthropomorphize, or attribute human characteristics to robots (Woods et al., 2007). Some have suggested that the tendency to anthropomorphize inanimate objects is linked to the need to belong (Epley et al., 2007; Reich and Eyssel, 2013). In their study, Epley and colleagues hypothesized that the consequence of loneliness was related to a person's tendency to anthropomorphize robots. Thus, a lack of belonging among other people could lead individuals to forge a connection with a non-living object (Epley et al., 2008). However, other investigations have not supported this assertion. For example, in a study on anthropomorphism, Ruijten et al. (2015) found that when playing an online, teamwork-oriented game, participants placed in a group where they were socially *included*, tended to anthropomorphize a robot more than those who were in a group where they were socially *excluded*. This would indicate an opposite effect than Epley's—that social inclusion rather than loneliness was linked to the identified tendency to anthropomorphize robots. The common factor is that in both cases social engagement did have a relationship with a person's tendency to anthropomorphize. Extroverts, in preferring company, would both be more prone to loneliness and more prone to the need to belong to a group.

When users see a robotic agent as anthropomorphic, they may also attribute specific personality traits to it. These attributions may also be related to the user's personality. Such personality attributions to robots can be compared to the way people attribute different personality traits to other humans (Woods et al., 2007). However, the findings related to the personality attribution in HRI are inconsistent. In a recent study, researchers collected personality ratings from participants, and then those participants rated a robots' personality traits (de Graaf and Ben Allouch, 2014). They found that the more extroverted the participant was, the more likely they were to attribute extroversion to the robot, but only in the cases where they already had high expectations of their robot-centered interaction. Conversely, a study where participants' personality traits were compared to their ratings of robot personality traits, found that people did not view themselves and the robot as having similar traits, regardless of obvious similarities found via a survey (Woods et al., 2005). It is possible that when a user does not see a robot as similar enough to a human, they never take the step of assigning the same type of personality attributions that they would assign to another person. In this way, assigning a personality to robots, would be related to the robots' anthropomorphic qualities.

In addition to anthropomorphism, robot likeability may also be influenced by the users' own personality traits. If users view their interactions with robots in a manner similar to the way they view their human-human interactions, extroverts may derive a more positive affect from the experience than introverts would (Watson and Clark, 1997). Even if they do not, the current body of findings on personality still supports the expectation of a more positive response to a robotic interaction for extroverts. People who are more extroverted generally find other entities more likable. In a study involving anonymous email users, participants who scored higher on extroversion tended to rate unseen correspondent partners as more likable (Byron and Baldridge, 2007). Although a key feature of extroversion is taking pleasure in interaction, as well as the tendency to enjoy interactions with both inanimate and animate entities, this does not exclude the important roles other variables might play, such as robot likeability in terms of how robots are viewed.

Anthropomorphism also influences emotional attachment, a key facet to long term repeated interactions. A recent study found that people form a stronger emotional attachment to products which they perceive as being similar to themselves (Govers and Mugge, 2004). Similarly, a study on individuals' responses to advertisements found that the more extroverted a person considered their ideal self to be, the more positively they rated the products in advertisements they viewed (Chang, 2001). If such personality effects can be extended to robots, then the more anthropomorphic or human-like someone perceives a robot to be, the more likely they are to form a bond with it and to find it likable.

The findings discussed above have come from disparate fields and varied studies. To date, there is no overarching, specification which determines exactly what facets of a robot lead people to like and/or anthropomorphize it. Since the literature on the relationship between personality traits and different aspects of HRI is presently underspecified, our present work seeks to clarify the relationship between participant extroversion and the tendency to anthropomorphize various types of robots.

### Current Exploration

In order to explore the relationship between a person's level of extroversion and their tendency to both like and anthropomorphize robots, we measured personality, including extroversion, and asked participants to rate robots on anthropomorphism and likability after they had observed robots completing job tasks and learned about their respective capabilities.

While previous research has resulted in unspecific findings, many studies have consistently confirmed that a person's tendency to anthropomorphize is related to their feelings about relationships with other humans (Epley et al., 2007, 2008; Woods et al., 2007; Ruijten et al., 2015). Whether extroverts connect with non-human entities in a similar way than they make connections with other humans, or if they simply view robots as non-living communicative tools, the same trait that drives them to interact with other humans may be beneficial to their interactions with robots. Extroverts can be expected to experience more successful interactions with robots, both in terms of perceiving human aspects of these robots and finding positive qualities to connect to the robot. Given that personality tends to be stable across situations, it can be argues that it is likely that extroversion, as a relatively fixed trait, leads to the positive ranking of likability and anthropomorphism of robots. For these reasons, we predict that extroversion will be positively related to two main outcomes:

1. A participant's level of extroversion will be a positive predictor of their anthropomorphization of a robot.
2. A participant's extroversion will be a positive predictor of how much they express their like for the candidate robot.

## Method

### Participants

Participants (*n* = 486) were selected from a student population. Participants were predominately undergraduates at the University of Central Florida in Orlando, Florida. They ranged in age from 18 to 50 (*M* = 20.06, *SD* = 3.819). All participants were recruited through SONA, an online system for managing research participation. Participants had the opportunity to receive class credits in exchange for their effort. All participants included answered the necessary surveys. Blank questionnaires were discarded and were not included in our final *n* of 486.

### Materials

The relevant questionnaires were deployed online through Qualtrics, a purpose-employed survey engine. Questionnaires consisted of a demographics survey, the Mini International Personality Item Pool (Mini-IPIP; Donnellan et al., 2006). The latter measures the “Big Five” personality traits of extroversion, agreeableness, conscientiousness, neuroticism, and intellect/imagination. We also used the Godspeed scale, which allows participants to rate robots on anthropomorphism, animacy, likableness, perceived intelligence, and perceived safety (Bartneck et al., 2009).

Additionally, the Negative Attitude Toward Robotics Scale (NARS; Nomura et al., 2006) was included, which measures participants' negative opinions on three separate subscales (Negative Attitudes toward Situations and Interactions with Robots; Negative Attitudes toward Social Influence of Robots; and Negative Attitudes toward Emotions in Interaction with Robots).

In addition to these scales, the study presented images and videos of human and robot job performers in each work condition. Here, we showed two job options (i.e., warehouse technician and an IED technician), and two job performers (i.e., human and robot). This yielded a total of four scenarios, as each job was performed by both the human and the robot (i.e., human IED technician, human warehouse technician, robot IED technician, and robot warehouse technician). Each scenario was depicted in an image, as well as a video of the human or a robot completing each required task. The video was a 1-min video montage, which included various clips of the job performers completing some tasks required for each job, with text describing the job overlaying the video. Videos for both the human and robotic job performers in the IED technician role depicted an agent searching for IEDs in a desert-type environment. In a similar fashion, human and robot warehouse technician videos depicted an agent retrieving items from a warehouse. While human and robot videos necessarily differed, the tasks they performed were equivalent. Scenarios involving human job performers were provided to participants, to establish a comparative baseline, but these were not evaluated here directly as they are were not the focus of the present work. The text overlay description was the same for the human and robotic job performer in each task completed. The image used for the warehouse robot was a picture of a Fetch by Fetch Robotics, which is a boxy white and blue model designed for use in warehouses. The image used for the IED robot was a TALON robot, manufactured by Foster-Miller. This is a military robot with large wheels, and one dexterous extendable arm.

### Design

Our procedure used a within-participant design employing two (human/robot) by two (IED technician/warehouse technician) viewing conditions. Each participant saw all four of these scenarios. The order in which these scenarios were presented was randomized across participants for counterbalancing purposes.

### Procedure

After committing to their participation via SONA, participants were directed to the Qualtrics website where the study was deployed. After reading and completing informed consent, participants completed the pre-experimental surveys, which consisted of the demographics survey, theMini-IPIP, and the NARS. They then began the viewing trials. Each trial started with a picture of a job performer (either human or robot); a video showing the human or robot doing various tasks involved in the job, and a paragraph describing the job requirements. Finally, participants filled out the Godspeed Scale to indicate their impressions of the job performer's traits.

## Results

### Correlational Matrices

First, participant scores on the NARS were correlated with their personality scores on the IPIP. As the NARS measures attitudes, and was administered prior to exposure to the videos, the relationships between these variables explain participants general negative attitudes toward robots in all situations, and not specifically in the manipulation of the robot or human job performer (see Table 1). All correlations were significant at *p* < 0.001.

**Table 1:** Correlations between the NARS and the IPIP.

	**1**	**2**	**3**	**4**	**5**	**6**	**7**	**8**
(1) Intellect	1							
(2) Conscientiousness	0.544	1						
(3) Extroversion	0.453	0.497	1					
(4) Agreeableness	0.686	0.586	0.562	1				
(5) Neuroticism	0.324	0.338	0.471	0.402	1			
(6) NARS (interaction)	0.240	0.381	0.538	0.395	0.517	1		
(7) NARS (social)	0.367	0.418	0.552	0.508	0.525	0.742	1	
(8) NARS (emotional)	0.196	0.280	0.257	0.214	0.283	0.439	0.411	1

The correlations between participant extroversion and the NARS, for social situations and interactions, were moderate [*r*_(485)_ = 0.552, and *r*_(485)_ = 0.538, respectively]. In fact, these two aspects of the NARS had a stronger relationship with extroversion than any of the other personality factors. Overall, agreeableness had the lowest correlations with the three NARS subscales [interaction *r*_(485)_ = 0.240, Social *r*_(485)_ = 0.367, and Emotional *r*_(485)_ = 0.196].

Correlations between participant personality traits (intellect, conscientiousness, extroversion, agreeableness, and neuroticism) and robot characteristics (anthropomorphism and likability) were calculated and are displayed in Table 2. While some of the values presented are not part of the present analysis of interest (that is, examining the precursors to the tendency to anthropomorphize), such variables are displayed for comparative purposes. All correlational values in the matrix provided are significant at *p* < 0.001.

**Table 2:** Correlations between godspeed scales and IPIP.

	**1**	**2**	**3**	**4**	**5**	**6**	**7**	**8**	**9**	**10**
(1) Intellect	1									
(2) Conscientiousness	0.544	1								
(3) Extroversion	0.453	0.497	1							
(4) Agreeableness	0.686	0.586	0.562	1						
(5) Neuroticism	0.324	0.338	0.471	0.402	1					
(6) Anthropomorphism	0.215	0.252	0.338	0.244	0.275	1				
(7) Animacy	0.304	0.284	0.323	0.307	0.243	0.576	1			
(8) Likability of	0.444	0.416	0.437	0.465	0.266	0.635	0.573	1		
(9) Perceived safety	0.451	0.430	0.428	0.452	0.185	0.539	0.503	0.815	1	
(10) Perceived intellect	0.415	0.386	0.366	0.441	0.253	0.427	0.669	0.659	0.657	1

While a great deal more data was correlated for this study, only the variables of interest are displayed. For the other correlations or individual correlations broken down by job performer, see Table 3. Table 1 shows the relationships between the personality variables of the participants and their subsequent rankings of the robots on the Godspeed scales. In order to determine the precursors of anthropomorphism and liking of the robots, all personality factors examined via the IPIP were considered. However, the trait of extroversion was the main focus since the theoretical foundation provided support that this trait was perhaps the most relevant predictor. The bivariate correlation confirmed our first hypothesis that extroversion is positively related to anthropomorphism. This pair was found to be the highest correlation between any participant personality variable and subsequent rating of the anthropomorphism of the robots, in the scope of this study [*r*_(485)_ = 0.338, *p* < 0.001]. However, the correlations between other personality variables and robot likeability ratings, with both agreeableness [*r*_(485)_ = 0.444, *p* < 0.001] and intellect (*r* = 0.465, *p* < 0.001) showed a higher correlation than between extroversion and the likability of robots [*r*_(485)_ = 0.437, *p* < 0.001]. This indicates that our initial hypothesis that extroversion is a positive predictor for the tendency to anthropomorphize, is supported. However, other personality variables do have to be taken into account when predicting whether someone will (a) anthropomorphize or (b) like a robot.

**Table 3:** Total Correlation Table.

		**1**	**2**	**3**	**4**	**5**	**6**	**7**	**8**	**9**	**10**	**11**	**12**	**13**	**14**	**15**	**16**	**17**	**18**	**19**	**20**	**21**	**22**	**23**	**24**	**25**	**26**	**27**	**28**	**29**	**30**	**31**	**32**	**33**	**34**	**35**	**36**	**37**
1	IPIP: Intellect	1																																				
2	IPIP: Conscientiousness	0.54	1																																			
3	IPIP: Extroversion	0.45	0.50	1																																		
4	IPIP: Agreeableness	0.69	0.59	0.56	1																																	
5	IPIP: Neuroticism	0.32	0.34	0.47	0.40	1																																
6	NARS: Interaction	0.24	0.38	0.54	0.40	0.52	1																															
7	NARS: Social	0.37	0.42	0.55	0.51	0.53	0.74	1																														
8	NARS: Emotional	0.20	0.28	0.26	0.21	0.28	0.44	0.41	1																													
9	Anthropomorphism: IED Human	0.46	0.41	0.40	0.47	0.21	0.31	0.39	0.16	1																												
10	Animacy: IED Human	0.47	0.42	0.40	0.47	0.21	0.30	0.38	0.15	0.96	1																											
11	Likability: IED Human	0.42	0.45	0.40	0.49	0.24	0.34	0.37	0.18	0.82	0.83	1																										
12	Perceived Intelligence: IED Human	0.52	0.47	0.42	0.52	0.25	0.31	0.40	0.19	0.87	0.90	0.87	1																									
13	Perceived Safety: IED Human	0.42	0.46	0.41	0.44	0.23	0.31	0.35	0.19	0.71	0.73	0.75	0.77	1																								
14	Anthropomorphism: Warehouse Human	0.44	0.39	0.40	0.48	0.25	0.30	0.39	0.23	0.77	0.78	0.69	0.76	0.63	1																							
15	Animacy: Warehouse Human	0.44	0.40	0.41	0.48	0.25	0.32	0.39	0.22	0.76	0.77	0.69	0.77	0.62	0.97	1																						
16	Likability: Warehouse Human	0.37	0.42	0.42	0.48	0.27	0.36	0.40	0.23	0.73	0.73	0.75	0.74	0.62	0.87	0.88	1																					
17	Perceived Intelligence: Warehouse Human	0.42	0.43	0.41	0.49	0.26	0.34	0.38	0.23	0.77	0.78	0.75	0.80	0.67	0.90	0.91	0.92	1																				
18	Perceived Safety: Warehouse Human	0.42	0.43	0.42	0.47	0.23	0.35	0.36	0.23	0.72	0.72	0.70	0.73	0.73	0.82	0.82	0.83	0.86	1																			
19	Anthropomorphism: IED Robot	0.22	0.25	0.34	0.24	0.28	0.34	0.25	0.18	0.30	0.30	0.38	0.33	0.44	0.30	0.30	0.32	0.32	0.38	1																		
20	Animacy: IED Robot	0.31	0.31	0.39	0.35	0.26	0.30	0.30	0.15	0.46	0.47	0.49	0.50	0.50	0.42	0.44	0.43	0.45	0.48	0.81	1																	
21	Likability: IED Robot	0.44	0.42	0.44	0.47	0.27	0.30	0.32	0.15	0.64	0.66	0.67	0.69	0.64	0.62	0.64	0.63	0.65	0.63	0.62	0.74	1																
22	Perceived Intelligence: IED Robot	0.45	0.41	0.44	0.51	0.27	0.29	0.37	0.16	0.72	0.74	0.71	0.76	0.64	0.66	0.67	0.64	0.69	0.65	0.51	0.65	0.83	1															
23	Perceived Safety: IED Robot	0.45	0.43	0.43	0.45	0.19	0.29	0.35	0.16	0.69	0.72	0.67	0.73	0.76	0.63	0.64	0.62	0.66	0.72	0.51	0.64	0.82	0.81	1														
24	Anthropomorphism: Warehouse Robot	0.25	0.25	0.32	0.29	0.25	0.28	0.21	0.17	0.38	0.36	0.42	0.39	0.42	0.43	0.42	0.43	0.44	0.49	0.62	0.57	0.53	0.46	0.45	1													
25	Animacy: Warehouse Robot	0.30	0.28	0.32	0.31	0.24	0.27	0.25	0.16	0.46	0.45	0.48	0.48	0.47	0.52	0.52	0.49	0.53	0.55	0.58	0.66	0.59	0.53	0.52	0.80	1												
26	Likability: Warehouse Robot	0.42	0.37	0.36	0.41	0.27	0.22	0.26	0.13	0.60	0.61	0.60	0.62	0.55	0.70	0.71	0.68	0.71	0.71	0.45	0.56	0.72	0.64	0.66	0.65	0.73	1											
27	Perceived Intelligence: Warehouse Robot	0.42	0.39	0.37	0.44	0.25	0.23	0.30	0.18	0.67	0.68	0.65	0.71	0.60	0.76	0.77	0.75	0.80	0.73	0.39	0.52	0.68	0.75	0.67	0.59	0.68	0.85	1										
28	Perceived Safety: Warehouse Robot	0.43	0.41	0.37	0.43	0.18	0.22	0.26	0.18	0.67	0.68	0.65	0.69	0.69	0.74	0.73	0.72	0.75	0.83	0.37	0.49	0.66	0.64	0.75	0.57	0.63	0.81	0.80	1									
29	Perceived Intelligence: (Robots Overall)	0.42	0.39	0.37	0.44	0.25	0.23	0.30	0.18	0.67	0.68	0.65	0.71	0.60	0.76	0.77	0.75	0.80	0.73	0.39	0.52	0.68	0.75	0.67	0.59	0.68	0.85	1.00	0.80	1								
30	Perceived Intelligence: (Humans Overall)	0.52	0.47	0.42	0.52	0.25	0.31	0.40	0.19	0.87	0.90	0.87	1.00	0.77	0.76	0.77	0.74	0.80	0.73	0.33	0.50	0.69	0.76	0.73	0.39	0.48	0.62	0.71	0.69	0.70	1							
31	Perceived Safety: (Robots Overall)	0.45	0.43	0.43	0.45	0.19	0.29	0.35	0.16	0.69	0.72	0.67	0.73	0.76	0.63	0.64	0.62	0.66	0.72	0.51	0.64	0.82	0.81	1.00	0.45	0.52	0.66	0.67	0.75	0.66	0.74	1						
32	Perceived Safety: (Humans Overall)	0.42	0.46	0.41	0.44	0.23	0.31	0.35	0.19	0.71	0.73	0.75	0.77	1.00	0.63	0.62	0.62	0.67	0.73	0.44	0.50	0.64	0.64	0.76	0.42	0.47	0.55	0.60	0.69	0.62	0.81	0.79	1					
33	Likability: (Robots Overall)	0.44	0.42	0.44	0.47	0.27	0.30	0.32	0.15	0.64	0.66	0.67	0.69	0.64	0.62	0.64	0.63	0.65	0.63	0.62	0.74	1.00	0.83	0.82	0.53	0.59	0.72	0.68	0.66	0.66	0.70	0.82	0.68	1				
34	Likability: (Humans Overall)	0.42	0.45	0.40	0.49	0.24	0.34	0.37	0.18	0.82	0.83	1.00	0.87	0.75	0.69	0.69	0.75	0.75	0.70	0.38	0.49	0.67	0.71	0.67	0.42	0.48	0.60	0.65	0.65	0.65	0.89	0.70	0.80	0.68	1			
35	Animacy: (Robots Overall)	0.30	0.28	0.32	0.31	0.24	0.27	0.25	0.16	0.46	0.45	0.48	0.48	0.47	0.52	0.52	0.49	0.53	0.55	0.58	0.66	0.59	0.53	0.52	0.80	1.00	0.73	0.68	0.63	0.67	0.47	0.50	0.47	0.57	0.45	1		
36	Animacy: (Humans Overall)	0.44	0.40	0.41	0.48	0.25	0.32	0.39	0.22	0.76	0.77	0.69	0.77	0.62	0.97	1.00	0.88	0.91	0.82	0.30	0.44	0.64	0.67	0.64	0.42	0.52	0.71	0.77	0.73	0.75	0.77	0.66	0.67	0.62	0.71	0.50	1	
37	Anthropomorphism: (Robots Overall)	0.22	0.25	0.34	0.24	0.28	0.34	0.25	0.18	0.30	0.30	0.38	0.33	0.44	0.30	0.30	0.32	0.32	0.38	1.00	0.81	0.62	0.51	0.51	0.62	0.58	0.45	0.39	0.37	0.43	0.38	0.54	0.46	0.64	0.41	0.58	0.33	1
38	Anthropomorphism: (Humans Overall)	0.46	0.41	0.40	0.47	0.21	0.31	0.39	0.16	1.00	0.96	0.82	0.87	0.71	0.77	0.76	0.73	0.77	0.72	0.30	0.46	0.64	0.72	0.69	0.38	0.46	0.60	0.67	0.67	0.66	0.88	0.71	0.77	0.67	0.84	0.44	0.77	0.37
	Mean	14.27	13.01	10.99	14.55	10.51	14.83	15.16	12.23	19.02	23.50	17.57	19.78	9.82	19.19	23.4	17.9	18.69	10.03	9.36	13.61	14.97	17.39	9.86	9.60	13.33	15.07	16.85	9.63	17.12	19.23	9.74	9.92	15	17.7	13.47	23.44	9.48
	SD	4.23	4.11	3.09	4.20	3.73	5.40	4.96	7.00	7.33	8.85	6.68	7.09	3.74	7.48	9.10	7.14	7.18	3.82	5.03	6.44	5.74	6.65	3.79	4.94	6.52	6.35	6.86	3.88	6.76	7.15	3.84	3.78	6.05	6.92	6.48	8.98	4.98

Additional correlations allow for more in-depth comparisons between human personality variables and various aspects of both the robots and the humans. While those correlations are found in the Table 3, some will be further expanded upon here. The relationship between extroversion and likability of robots was stronger *r*_(485)_ = 0.437 than the relationship between extroversion and likability of the humans *r*_(485)_ = 0.404. This is not to say that extroverts considered the robots more likable than the humans, but that participant extroversion was a stronger predictor of whether or not they would like the robots, rather than whether or not they would like the human job performers.

When broken down by job type, the responses to the robots followed similar but not identical patterns. The highest correlations between likability of the IED robot and participant personality were agreeableness *r*_(485)_ = 0.465, and openness *r*_(485)_ = 0.444. Likability of the Warehouse robot similarly correlated strongly with participant agreeableness *r*_(485)_ = 0.441, and openness *r*_(485)_ = 0.415. When examining anthropomorphism of the IED robot, the strongest correlations were with extroversion *r*_(485)_ = 0.338, and neuroticism *r*_(485)_ = 0.275. For the Warehouse robot, the strongest correlations were extroversion *r*_(485)_ = 0.316 and agreeableness *r*_(485)_ = 0.290.

### Regressions and Bayesian Significance Modeling

A Bayesian regression analysis was also conducted on the data. This analysis was enacted in order to explore not only the coefficients, but also to demonstrate to what extent the plausibility of the alternative hypothesis put forward earlier, exceeds the probability of the null hypothesis. Specifically, the data were analyzed using the Jeffrey's Awesome Statistical Program (JASP) in order to determine the Bayes Factors and the best model for predicting each of the dependent variables. For both the prediction of robot anthropomorphism and robot likability, the best model for predicting the robot variable was the one including the same identified human personality antecedents as expressed in the prior regression.

Data were analyzed using the Jeffrey's Awesome Statistical Program (JASP) which applied the Bayesian analysis. The Bayesian regression analysis was used to determine the model which best predicts the data. Measured personality variables of the participant were regressed on their ratings of the robots, on the variables of interest, in order to predict which personality profile best predicted their ratings on anthropomorphism and likability.

#### Anthropomorphism

The regression results included only significant predictors of the dependent variable. Any predictor variable that was not significant at the *p* < 0.05 level was not included in the final regression model. For this reason, not all five of the surveyed personality traits were included. The regression of anthropomorphization on conscientiousness, extroversion, and neuroticism was significant, *F*_(3, 482)_ = 30.81, *p* < 0.001. The adjusted *R*^2^ value of 15.6% is low but not negligible in determining the reasons why some individuals may anthropomorphize a robot. The personality factors of extroversion, neuroticism, and conscientiousness predict an important portion of the variation of the tendency to anthropomorphize. The full regression equation being:

$$\begin{array}{l} Y_{\text{anthropomorphization}}\;=\;2.266+0.1229X_{\text{conscientiousness}}\\ +0.3496X_{\text{extroversion}}+0.1692X_{\text{neuroticism}} \end{array}$$

The model was also examined after controlling for the impact of the NARS. Through a hierarchical regression, the three subscales of the NARS were controlled to determine the impact of the predictors, after accounting for whatever negative attitudes a participant might already have toward robots. The change in *R*^2^ was 0.051. This indicates that a significant amount of the variance in anthropomorphism predicted by a participant's extroversion, could also be predicted by the NARS alone. However, about 5% of a person's tendency to anthropomorphize still relies upon their extroversion. In the model in which the NARS is accounted for, extroversion was the only personality variable that was a significant predictor of anthropomorphism, with a beta coefficient of *b* = 0.282.

#### Likability

The regression of likability on intellect, conscientiousness, extroversion and agreeableness proved significant [*F*_(4, 481)_ = 51.68, *p* < 0.001] An adjusted R^2^ value of 29.5% showed that personality variables were responsible for an important degree of the variance in likeability. The full regression equation is:

$$\begin{array}{l} Y_{\text{likability}}\;=\;2.216+0.2805X_{\text{intellect}}+0.1721X_{\text{conscientiousness}}\\ +0.3402X_{\text{extroversion}}+0.1938X_{\text{agreeableness}} \end{array}$$

When the results from the NARS were controlled, the change in *R*^2^ became 0.185. The same predictor variables of intellect, conscientiousness, extroversion, and agreeableness were significant in predicting the extent to which someone will like a robot; however, the lower *R*^2^ indicates that these variables only predict 18.5% of the variance beyond that which has already been predicted by the NARS.

#### Bayesian Significance

The level of robot anthropomorphization was best predicted by a model containing the factors concerning participant's conscientiousness, extroversion and neuroticism. This produced a Bayes Factor with overwhelming evidence that the model was a better predictor of anthropomorphization than the null model (B_10_ = 1.584 × 10^15^). The likability of the robot was best predicted by considering the participant's level of intellect, conscientiousness, extroversion, and agreeableness. This exhibited a similarly high Bayes Factor (B_10_ = 8.978 × 10^32^). Both high values indicate the extent to which the specified model represents a better predictor of the data than chance would alone.

## Discussion

We have sought to explore the relationship between personality variables of an individual and their predictive validity in anticipating their perceptions of both anthropomorphism and likeability of robots. Our first hypothesis posited that extroversion would be the strongest predictor of the tendency to both like and anthropomorphize robots. This expectation was supported, as expressed in the significant correlations, indicating a moderating relationship between participant extroversion and the post-interaction ratings of both anthropomorphism and likeability. We should also note that several other personality factors were also found to be influential. A Bayesian analysis was used to establish the model which best predicted the participant's ratings of the robots, based on their personality variables. Neuroticism played a secondary role to extroversion in predicting anthropomorphizing. Conscientiousness was also an important contributor. When it came to predicting likability of robots, agreeableness and intellect were both stronger predictors than extroversion. Conscientiousness again added to the model. While the models were expanded beyond our initial hypothesis, there is no denying that extroversion plays a critical role. These results support both Ruijten et al. (2015) and Epley et al.'s (2008) contentions that social inclusion and the need to belong can influence a person's tendency to anthropomorphize. Here, we extend this assertion to HRI and confirmed Byron and Baldridge's (2007) notion that extroverts have a more positive reaction overall to other entities. The fact that the likability ratings were even more highly correlated with extroversion then with anthropomorphism, both supports previous observations about the nature of extroversion, and provides a possible explanation of why different individuals may have very different reactions to the same robot. In HRI research, robot antecedents of interaction have been examined much more frequently than human differences. Our findings about the influence of personality on the perceptions of robots, was consistent with some previous findings that individual differences play a large role in people's reactions to robots, regardless of operational context.

In examining the data from the NARS, there were some unexpected findings. The NARS was administered before the scenarios involving the job performers, and therefore measured a person's attitudes toward robots in general and was not affected by the particular robot stimuli used. It was unexpected that extroverts would have higher negative attitudes toward robots, on the NARS, when extroversion was later one of the significant predictors of how much they liked the robots. It is certainly possible that the abstract idea of robots might be off-putting to a social person, but the actual robot stimuli was not bothersome in the same way.

The issue of robot morphology and the role it plays in mediating human perception of robots, cannot be discounted in any way. This study examined only two different robot forms, but it is important to consider the possibility that there may be an interaction effect between robot appearance and robot job performance. In other words, a person might respond very differently to two robots of differing appearance, even if they worked in the same job domain. This could mean that such an experiment as outlined here, replicated using different robots, might yield different results.

### Practical Implications

We have reported statistically significant ways to form predictions based on human personality variables and predicting both the likability and anthropomorphizing of robots. Additionally, by using Bayesian statistical modeling, we have found that the regression models including certain personality characteristics are overwhelmingly more likely to be predictors of the obtained results, than chance alone. Thus, we propose that an individual's personality is an important factor associated with a propensity to anthropomorphize or like robots. This may be particularly useful for organizations planning to add robots to their workplaces, since it will be beneficial to assess the personality of their workforce to help predict the acceptance of such robots. These results also have implications for robot designers who may leverage this to better comprehend user personality influences and to understand how levels of anthropomorphism could be leveraged in different ways for varying types of robots.

### Future Research

It is critical to understand how different people respond to robots in a variety of settings. While this study included only workplace settings in which robots are already integrated, such as factories and IED disposal, robot penetration to many different spheres is fast approaching. Robots are already used for home cleaning and organization as well as being integrated into numerous medical fields. Predicting how professionals and communities will respond to robot interaction must be understood early in order to help users transition to these new arrangements.

Future research should evaluate people who hold positions similar to the those robots will be completing. For instance, warehouse workers rather than a general college sample will be needed to evaluate a warehouse robot, and perhaps someone who cleans in their home should be the population who evaluates systems used for automatic housework. Such individuals could perform a similar analysis of robots working in their own field and differing fields to determine whether, when controlling for individual levels of extroversion, a more stereotypically extroverted population would be more accepting of robotic counterparts.

IN addition, developers will need to understand the ways in which negative responses to robots can be mitigated. Future studies can employ robotic images of varying levels of anthropomorphism (humanlike, somewhat humanlike, and non-humanlike), to determine if the negative introversion-anthropomorphism link can be offset through design. Namely, can you overcome an introvert's lack of anthropomorphization, by making the robot more humanlike? Perhaps the lack of anthropomorphizing an entity is a property inherent to all introverts. A deeper understanding of that relationship can inform both those who seek to develop robots, and those who seek to integrate them into our modern world.

## Data Availability Statement

The raw data supporting the conclusions of this manuscript will be made available by the authors, without undue reservation, to any qualified researcher.

## Ethics Statement

This study was carried out in accordance with the recommendations of the University of Central Florida Institutional Review Board with written informed consent from all subjects. All subjects gave written informed consent in accordance with the Declaration of Helsinki. The protocol was approved by the UCF Institutional review board.

## Author Contributions

All authors were involved in the process. TS was the main driving force in the data collection, AK did most of the analysis, while PH did a large amount of editing and additional writing.

### Conflict of Interest Statement

The authors declare that the research was conducted in the absence of any commercial or financial relationships that could be construed as a potential conflict of interest.

## References

[B1] Bartneck C. Kulić D. Croft E. Zoghbi S. (2009). Measurement instruments for the anthropomorphism, animacy, likeability, perceived intelligence, and perceived safety of robots. Int. J. Soc. Robot. 1, 71–81. 10.1007/s12369-008-0001-3

[B2] Byron K. Baldridge D. C. (2007). E-mail recipients' impressions of senders' likability: the interactive effect of nonverbal cues and recipients' personality. J. Bus. Commun. 44, 137–160. 10.1177/0021943606297902

[B3] Chang C. (2001). The impacts of personality differences on product evaluations. NA Adv. Consum. Res. 28, 26–33. Available online at: http://acrwebsite.org/volumes/8423/volumes/v28/NA-28

[B4] de Graaf M. Ben Allouch S. (2014). Expectation setting and personality attribution in hri, in Proceedings of the 2014 ACM/IEEE International Conference on Human-Robot Interaction, (Bielefeld: ACM), 144–145.

[B5] Donnellan M. B. Oswald F. L. Baird B. M. Lucas R. E. (2006). The mini-IPIP scales: tiny-yet-effective measures of the big five factors of personality. Psychol. Assess. 18, 192–203. 10.1037/1040-3590.18.2.192 16768595

[B6] Epley N. Akalis S. Waytz A. Cacioppo J. T. (2008). Creating social connection through inferential reproduction: loneliness and perceived agency in gadgets, gods, and greyhounds. Psychol. Sci. 19, 114–120. 10.1111/j.1467-9280.2008.02056.x 18271858

[B7] Epley N. Waytz A. Cacioppo J. T. (2007). On seeing human: a three-factor theory of anthropomorphism. Psychol. Rev. 114, 864–886. 10.1037/0033-295X.114.4.864 17907867

[B8] Eysenck H. J. (1950). Dimensions of Personality, Vol. 5. New Brunswick, NJ: Transaction Publishers.

[B9] Govers P. C. Mugge R. (2004). I love my Jeep, because it's tough like me: the effect of product-personality congruence on product attachment, in Proceedings of the Fourth International Conference on Design and Emotion (Ankara, Turkey).

[B10] Hancock P. A. Billings D. R. Schaefer K. E. Chen J. Y. De Visser E. J. Parasuraman R. (2011). A meta-analysis of factors affecting trust in human-robot interaction. Hum. Factors 53, 517–527. 10.1177/0018720811417254 22046724

[B11] Letheren K. Kuhn K. A. L. Lings I. Pope N. K. L. (2016). Individual difference factors related to anthropomorphic tendency. Eur. J. Market. 50, 973–1002. 10.1108/EJM-05-2014-0291

[B12] Nomura T. Kanda T. Suzuki T. (2006). Experimental investigation into influence of negative attitudes toward robots on human–robot interaction. AI Soc. 20, 138–150. 10.1007/s00146-005-0012-7

[B13] Nomura T. Kanda T. Suzuki T. Kato K. (2008). Prediction of human behavior in human–robot interaction using psychological scales for anxiety and negative attitudes toward robots. IEEE Trans. Robot. 24, 442–451. 10.1109/TRO.2007.914004

[B14] Parasuraman R. Riley V. (1997). Humans and automation: use, misuse, disuse, abuse. Hum. Factors 39, 230–253. 10.1518/001872097778543886

[B15] Reich N. Eyssel F. (2013). Attitudes towards service robots in domestic environments: the role of personality characteristics, individual interests, and demographic variables. Paladyn J. Behav. Robot. 4, 123–130. 10.2478/pjbr-2013-0014

[B16] Ruijten P. A. Midden C. J. Ham J. (2015). Lonely and susceptible: the influence of social exclusion and gender on persuasion by an artificial agent. Int. J. Hum. Comput. Interact. 31, 832–842. 10.1080/10447318.2015.1067480

[B17] Watson D. Clark L. A. (1997). Extraversion and its positive emotional core, in Handbook of Personality Psychology (San Diego, CA: Academic Press), 767–793.

[B18] Woods S. Dautenhahn K. Kaouri C. Boekhorst R. Koay K. L. (2005). Is this robot like me? Links between human and robot personality traits, in 2005 5th IEEE-RAS International Conference on Humanoid Robots (Amsterdam), 375–380.

[B19] Woods S. Dautenhahn K. Kaouri C. te Boekhorst R. Koay K. L. Walters M. L. (2007). Are robots like people?: relationships between participant and robot personality traits in human–robot interaction studies. Interact. Stud. 8, 281–305. 10.1075/is.8.2.06woo

